# A non-targeted data processing workflow for volatile organic compound data acquired using comprehensive two-dimensional gas chromatography with dual channel detection

**DOI:** 10.1016/j.mex.2020.101009

**Published:** 2020-07-24

**Authors:** Julianne M. Byrne, Lena M. Dubois, Jonathan D. Baker, Jean-François Focant, Katelynn A. Perrault

**Affiliations:** a Laboratory of Forensic and Bioanalytical Chemistry, Forensic Sciences Unit, Chaminade University of Honolulu, Honolulu, HI, USA; b Organic and Biological Analytical Chemistry Laboratory, MolSys, University of Liège, Liège, Belgium; c School of Natural Sciences and Mathematics, Chaminade University of Honolulu, Honolulu, HI, USA

**Keywords:** Flame ionization detection, Quadrupole mass spectrometry, Multidimensional chromatography, Simultaneous detection

## Abstract

There has been an influx of technology for comprehensive two-dimensional gas chromatography analyses in recent years, calling for development of guided workflows and rigorous reporting of processes. This research focuses on the processing method for data collected on a dual channel detection system using flame ionization detection (FID) and quadrupole mass spectrometry (qMS) for the analysis of volatile organic compounds (VOCs). The samples analyzed were kava (*Piper methysticum*), which has a rich VOC profile that benefits substantially from a multidimensional approach due to enhanced peak capacity. The procedure which was customized here was the data processing workflow from a manual single-sample analysis to an integrated batch workflow that can be applied across studies.

• Parameter choice for baseline correction and peak detection were defined when handling batch data.
• Elution regions were defined using qMS data to automate compound identification.
• Stencils were transformed onto FID data and sequenced for quantitative information.

This dataset can be used as a training tool, as all details, methods and results for the workflow have been provided for users to compare with. The focus on data workflow reproducibility in the field of multidimensional chromatography will assist in adoption by users in new application areas.

## Graphical abstract

**Figure:**
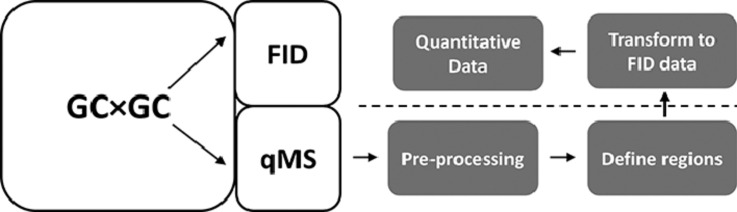

Specifications table

**Table:** 

Subject Area:	Chemistry
More specific subject area:	Separation Science, Chemometrics
Method name:	Data processing workflow for GC × GC-qMS/FID data
Name and reference of original method:	The instrument method was originally developed for VOC analysis in a pervious publication and this method paper focuses on building upon the data processing workflow. Reference: Dubois, L. M., Aczon, S., Focant, J.-F., Perrault, K. A., Translation of a one-dimensional to a comprehensive two-dimensional gas chromatography method with dual-channel detection for volatile organic compound measurement in forensic applications. Anal. Chem. 2020, 92, 10091–10098.
Resource availability:	All metadata, including raw data files and method are available as a published dataset in the Harvard dataverse, referenced in article. Byrne, J. M., Dubois, L. M., Baker, J. D., Focant, J.-F., Perrault, K. A., Methods for sampling, acquisition and data processing of six kava (Piper methysticum) samples collected and analyzed by GC × GC-qMS/FID. 2020, DOI: https://doi.org/10.7910/DVN/OUP5SW, Harvard Dataverse, V1.

## Method details

In recent years, there has been an influx of new technology in the field of comprehensive two-dimensional gas chromatography (GC×GC). These developments have come in various forms such as modulator options, detector coupling, simultaneous dual channel detection, ionization techniques, and software. This new range of available technology for GC×GC has had a multitude of effects. It has served to make GC×GC more accessible to the wider analytical community, leading to the implementation of GC×GC in a much broader range of applications. However, increasing complexity of data with new developments, such as dual channel detection, comes with higher complexity in how to handle large datasets with rich information coming from several sources. With many new users looking to implement GC×GC hardware and software, there is a strong need for clearer workflow descriptions and training tools for processing acquired data. There has also been significant dialogue amongst expert users in the field regarding the need for improved transparency in how data processing is performed. In order for GC×GC to be successfully adopted across many applications, data processing needs to be reproducible when different users or laboratories aim to obtain comparable results. Improved transparency and reproducibility in data processing could also afford an uptake in inter-laboratory validation, which is the next step in developing regulatory methods that rely on GC×GC.

This paper describes a method for batch processing of GC×GC data acquired from the headspace of kava (*Piper methysticum* G. Forst.). The samples were analyzed for the volatile organic compound (VOC) profile. Further details about the relevance of kava can be found in the “Additional Information” section of this article. The VOCs of each kava sample were collected using headspace solid-phase microextraction Arrow and were analyzed using GC×GC with dual channel detection by quadrupole mass spectrometry/flame ionization detection (qMS/FID). A reverse fill/flush (RFF) modulator was also used. An exhaustive list of all experimental parameters is provided in the supporting information, along with screenshots of each method application interface and exported method files from the analysis. The method describes the experimental parameters and data processing workflow used for the analysis. This is in contrast to manual workflows that are applied on single sample bases for a large majority of GC×GC analyses conducted in industry and research. The implications of different settings in the workflow are discussed throughout. The supporting data and all associated files used for this study are available in the Harvard Dataverse, so that users who are looking to become familiar with this workflow may reproduce the analysis as a training tool. This link includes all original raw data files [1], methods used for the acquisition and processing of the data [2], as well as the results of the analysis [3], [4] for readers to consult for comparison purposes. The dataset was kept intentionally small for this study to allow users to reproduce the analysis if desired without introducing unnecessary complexity. The instrument method was originally developed for VOC analysis in a pervious publication [5] and this method paper focuses on building upon the data processing workflow.

### SPME arrow sampling

Solid-phase microextraction Arrow sampling with a polydimethylsiloxane (PDMS)/carbon wide range (CWR) fiber (Restek Corporation, Bellefonte, PA, USA) was performed using 20 mL headspace vials (Restek Corporation) containing approximately 1.00 g of kava root powder. Six kava root powder samples were analyzed, including Tikaram's (Tonga), Unknown (Tonga), Tikaram's (Vanuatu), Mai (Fiji), Happy Warrior (Fiji), and Tikaram's Pure Waka (Fiji). Sample extraction and injection was performed using a TriPlus RSH™ Autosampler (Thermo Scientific, Waltham, MA, USA).

Sample incubation was performed at 35 °C at 500 rpm for 5 min. Sample extraction was performed at 35 °C at 1000 rpm for 5 min. The needle speed was 20 mm/s in the vial and the needle depth was set to standard. The incubation mode was constant. Injection was performed to a depth of 45 mm, with 35 mm/s penetration speed for 1 min. The GC was set to start when the needle entered the injector. SPME Arrow fibers were conditioned for 30 min at 260 °C prior to each sequence and were confirmed blank with a fiber blank injection. SPME Arrow fibers were reconditioned with a pre-desorb time of 2 min and a post desorb time of 5 min.

### GC×GC-qMS/FID method

The instrument configuration consisted of a Trace 1300 GC/FID and an ISQ 7000 Single Quadrupole Mass Spectrometer (Thermo Scientific). The inlet was in split mode with a split flow of 20 mL/min and a purge flow of 5 mL/min. The inlet temperature was 250 °C. The first dimension column was an Rxi-624Sil MS column (30 m × 0.25 mm ID × 1.4 µm film thickness, Restek Corporation) and the second dimension column was a Stabilwax (5 m × 0.25 mm × 0.25 µm film thickness, Restek Corporation). An INSIGHT Reverse Fill/Flush Modulator (SepSolve Analytical, Peterborough, UK) was used with a modulation period of 2.5 s and flush time of 100 ms. The loop dimensions were 0.53 mm ID × 1133 mm with a loop volume of 25 µL. The bleed line dimensions were 5 m × 0.1 mm ID. Ultra high purity helium (Airgas, Radnor, PA, USA) was used as the carrier gas. The flow rate in the first dimension column was 1.00 mL/min and the auxiliary gas flow rate was 20.00 mL/min, with a calculated flow rate of 17.9 mL/min in the second dimension. The flow was split between the FID and MS at approximately 4.5:1 which was maintained constant throughout the run. The GC oven started at 60 °C, was held for one minute, increased to a final temperature of 250 °C at the rate of 5 °C/min, and held for 10 min. The total run time was 49 min.

The ion source temperature and the transfer line temperature for the qMS were both set to 280 °C. The qMS was operated in electron ionization mode with a scan range from 40 to 300 *m/z.* The total scan time was 0.0241 s, which resulted in an acquisition rate of 41.5 scans/s. This is the maximum acquisition rate for this instrument when using this scan range. The FID was operated with 350 mL/min ultra-zero grade air (Airgas), 40 mL/min ultra high purity nitrogen as makeup gas (Airgas), and 35 mL/min ultra high purity hydrogen (Airgas). The temperature of the FID was set at 250 °C. The scan rate of the FID was 120 Hz. Instrument control was performed using Chromeleon 7 version 7.2.9 (Thermo Scientific).

### Data processing

**Fig. 1:**
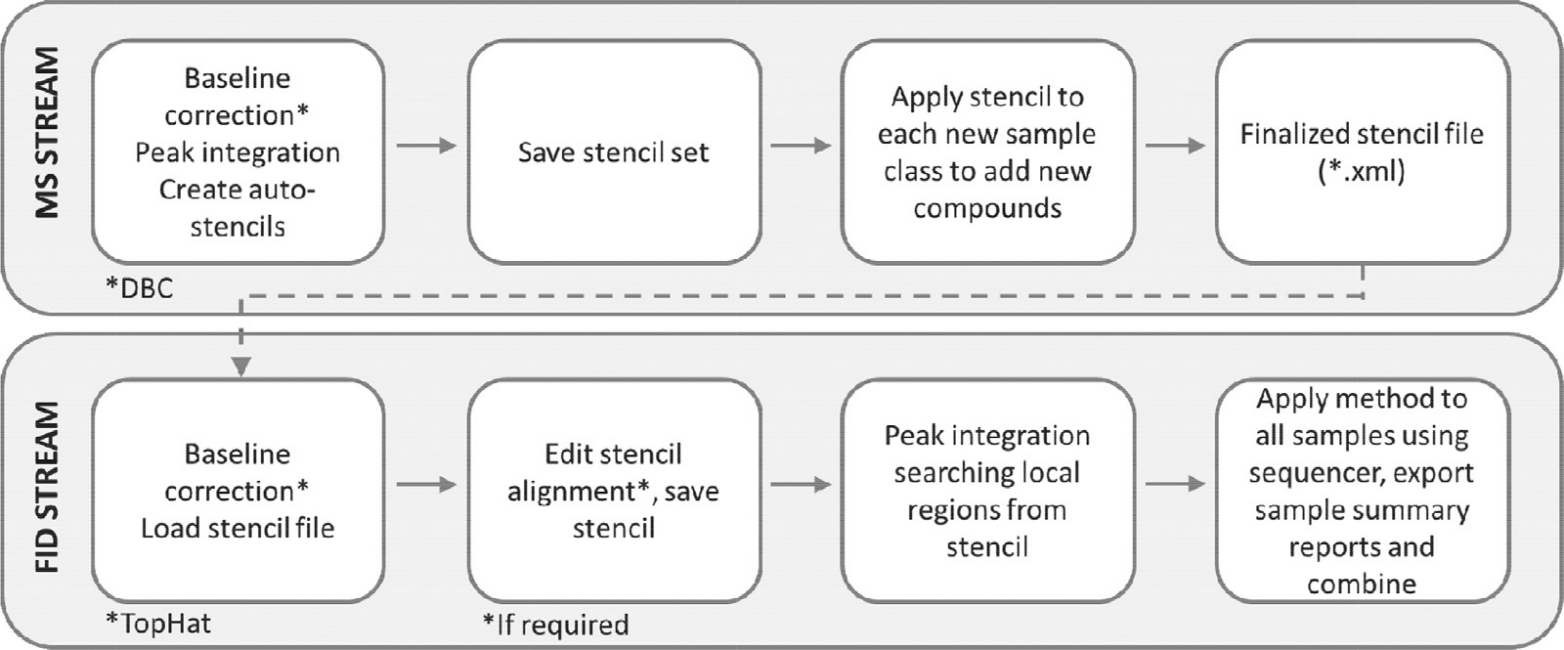
Data processing workflow for batch analysis of kava samples using GC × GC-qMS/FID.

A summary of the data processing protocol is provided in Fig. 1. Data acquired in Chromeleon was exported as *.raw files for qMS data, and *.cdf files for FID data. The qMS files were then converted to *.cdf in File Converter (Thermo Scientific). The *.cdf files were then imported into Chromspace v. 1.4 (SepSolve Analytical).  Any *.cdf files from other data acquisition software for different instrumentation manufacturers could also be imported at this stage. In Chromspace, the MS contour plots were used to tentatively identify compounds and create a set of stencils that was then transferred to the FID data for quantification. Baseline correction was performed using 0.35 s. All other details of the data processing parameters are listed in the supporting information.

The peak tables generated from the sequenced FID data were exported into a Microsoft Excel file (*.xlsx) to perform alignment of the compounds. The sample summary report for each sample was exported and zero values were added for any peaks that were not detected. The peak area column from individual sample summary reports were then combined (copied and pasted) into the same excel spreadsheet to ensure all data was combined in a single worksheet. This could also be accomplished using automated tools within the user's spreadsheet software. Further development of combined summary reports by software manufacturers would also assist making this process faster. Once the data in excel was transposed, it was exported into The Unscrambler X version 10.4 (CAMO Software, Oslo, Norway) to perform Principal Component Analysis (PCA). The data was treated by mean centering, scaling to standard deviation, and unit vector normalizing the peak table.

### Method considerations

Conventional manual data processing of these samples by using untargeted analysis would generate individual sample reports with a high number of peaks detected. The mean number of peaks detected by the qMS in the untargeted analysis of kava (*Piper methysticum*) was 349, and ranged from 314 to 384. However, the peaks identified across different samples are not necessarily consistent and this can cause major challenges in manually aligning this multivariate data in a reliable manner. This can mean that some peaks are misidentified as the same peak across samples, or that the same peak can be inappropriately labeled as multiple unique peaks across different samples. The new data processing workflow that involved the use of generated stencils allows for analysis of a lower number of peaks, filtering out instances of duplicate peak makers, as well as components present as instrument artifacts (e.g. column bleed). The use of this workflow allows tools that are typically exploited for targeted batch analyses to be used to extract the most meaningful peaks in an untargeted workflow, and to have them aligned reliably in a final aligned peak table. The use of a stencil that can be adjusted to samples with shifted retention times is also valuable, as it can be manually performed by transposing the stencil to the new area of interest, or alternatively performed using automated software tools for aligning individual chromatograms.

The generation of boundaries around peaks of interest for performing peak detection in multidimensional chromatography has been implemented in various software. It is fundamentally similar to the integration of peaks in conventional one-dimensional data, but applied to a two dimensional plot. In the case of one-dimensional data, most commercial software asks for a retention time window in which the peak metadata is searched. In the case of multidimensional chromatography, these regions have to exist in two retention windows along both axes, hence the need to define a specific region rather than a linear window. In different software, the regions are referred to using different terms such as stencils or templates. The software used in this study permitted auto-generation of stencil regions [6]. For MS data this includes metadata such as first dimension retention window, second dimension retention window, compound name, mass spectrum, ion ratios, etc. [7]. Other software have also implemented these types of region-defining approaches for multidimensional data, for example using a smart template concept [7], [8], [9]. When peak regions from the qMS data are defined, those regions can then be superimposed over the FID peak pattern, and the regions then represent the location and proposed identity of the component without associating a mass spectrum [6]. The multidimensional nature of the data makes the superimposing of this stencil pattern over peaks of interest in single channel output (e.g. FID) effective, as the regions can be aligned along both axes.

**Fig. 2:**
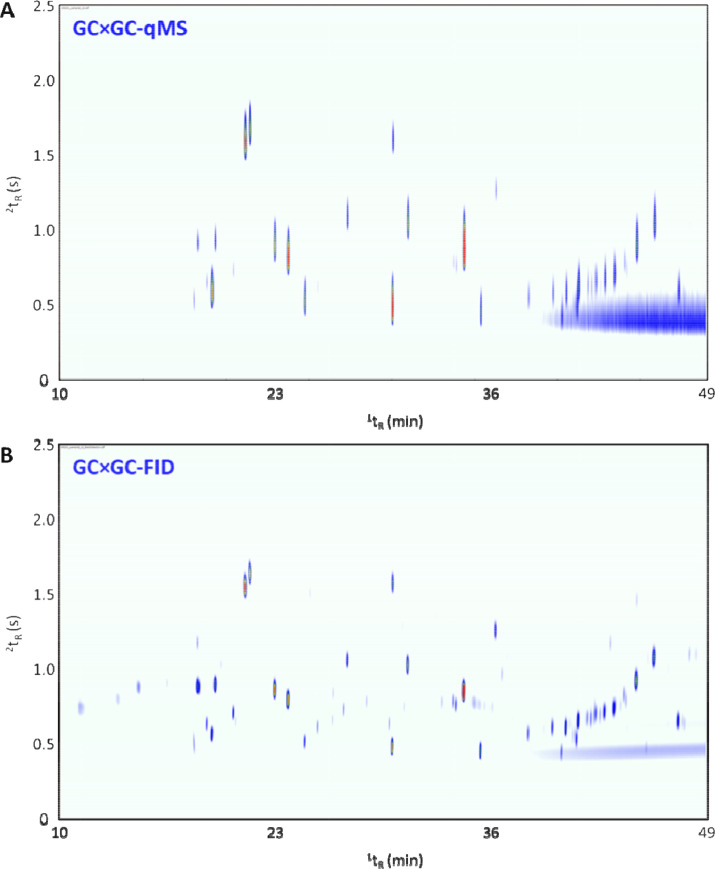
(A) Total ion current contour plot from Tonga kava (sample 2) generated using quadrupole mass spectrometry; (B) contour plot from Tonga kava (sample 2) generated using flame ionization detection.

The integration of two separate data streams has to be conducted mindfully. In the case of qMS/FID combination, the qMS stream was intended to provide structural information about compounds of interest. The qMS scan rate (e.g. 41.5 scans/s) was much lower than the FID acquisition rate (120 Hz). Therefore, in Fig. 2, it can be observed that the peak shape in the FID stream was vastly improved with reduced tailing. This has implications in terms of quantitative information, where small changes in VOC concentrations would likely be more effectively monitored in the FID stream than in the MS stream. This is due to improved integration on better peak shapes that experience less tailing. This means that it is also likely to provide improved monitoring of differences between sampling over monitoring fluctuations coming from noise, background, or chemical interferences. Depending on the split between the qMS and the FID detectors, the sensitivity can also be different between the two channels.

The initial processing involved baseline correction on the qMS data, which must be done differently in the qMS stream than in the FID stream. The dynamic baseline correction (DBC) algorithm was applied, which must take into account the second dimension (^2^D) peak width. This peak width has to be defined in most commercial software packages and can have a strong impact on the quality of baseline correction. In altering the peak width to different parameters, large differences were noted in final results. This is important because in batch VOC data from biological sources, dynamic range can span several orders of magnitude for compounds within a single sample, and also for a single compound across the entire batch of samples [10]. Therefore, in building the method, an average peak width had to be calculated and specified. The authors chose to average the peak width of 10 peaks spanning the entire chromatographic space that represented the dynamic range of one representative sample. This average took into account peaks that were smaller in concentration and also peaks which were approaching overloading, in order to represent the dynamic range that can be experienced across the data. The resulting peak width for baseline correction was finally set at 0.35 s, which means that this value had to be set as a compromise across the samples. There is not one value which would favor baseline correction across all samples and all peaks due to the high amount of variation and dynamic range across the samples.

After peak integration (settings defined in supporting information), regions were automatically assigned for peaks of interest using the software (i.e. autostencils) for the qMS data. Each region was also adjusted further to delineate each peak manually. At this stage, the file containing all regions (i.e. stencil file) was loaded on each sample sequentially, and new regions were added at each stage for compounds not previously detected by the integration algorithm on the previous samples. Herein, all compounds detected from all samples were included in the file that included these generated regions, which was then saved as a file for application to FID data. In large batch data with numerous replicates, it is impractical and time-consuming to go through this process on every single sample. In larger datasets, investigators may choose to select a single replicate sample from each class of data in the batch. For example, in a dataset containing 50 samples, in which 10 biological replicates exist across five sample classes, perhaps only one or two samples from a class need to be chosen for stencil creation. However, if an arbitrary sampling plan for stencil creation is chosen, this should be stated in the research methods, so that reasons for discrepancies in replication of results can be traced.

When applying the MS regions to FID data, the chromatogram file had to be baseline corrected. Most software uses different algorithms for baseline correction from the two different data streams (e.g. single channel vs. multichannel streams). In this particular study, FID data was baseline corrected using a TopHat algorithm that is designed for FID baseline correction. One major benefit of working on the corrected FID files rather than the qMS files is that this workflow is significantly less demanding on computing power; therefore, once the peak regions were defined using the MS stream, the further processing of the FID data was much faster for sequencing large batches. On small datasets such as the one presented herein, this is less of a concern. However, in larger batch datasets, for example those involving large number of replicates and subjects in biomedical research or for routine industrial analysis, this benefit is substantial for data processing. This therefore shifts the focus of the analyst on creating a high quality stencil for peaks of interest, which is likely to be the most laborious step in data processing when analyzing large batches of samples.

The application of the MS-produced stencil to FID may require slight shifting depending on the synchronization between the two streams, as the qMS is operating under vacuum and the FID under atmospheric pressure. Differences in peak width/quality can also contribute to the need to align regions of interest between different detection channels. However, the structure of the GC×GC data across the chromatographic plane is greatly beneficial in this scenario, as it provides a two-dimensional guide to shift the stencil into place. This eliminates the need for ensuring second dimension retention time alignment between both channels as the re-alignment of the stencil to the data takes only seconds. Peak searching can then be performed for the defined local regions from the stencil set and summary reports of each sample can be generated. These matrices can then be manually aligned into a single batch report for sample comparison using univariate or multivariate approaches. One should note that the combination of files to produce aligned data is a process which has differing results in quality across software platforms and often requires extensive manual verification; however, this step is crucial to batch data processing, which is a hindrance to the acceptance of GC×GC in fields requiring large batch analysis (e.g. metabolomics). The current practice is to manually align and combine batch data external to acquisition software in a spreadsheet program in order to generate the necessary format for external multivariate processing by other software.

**Fig. 3:**
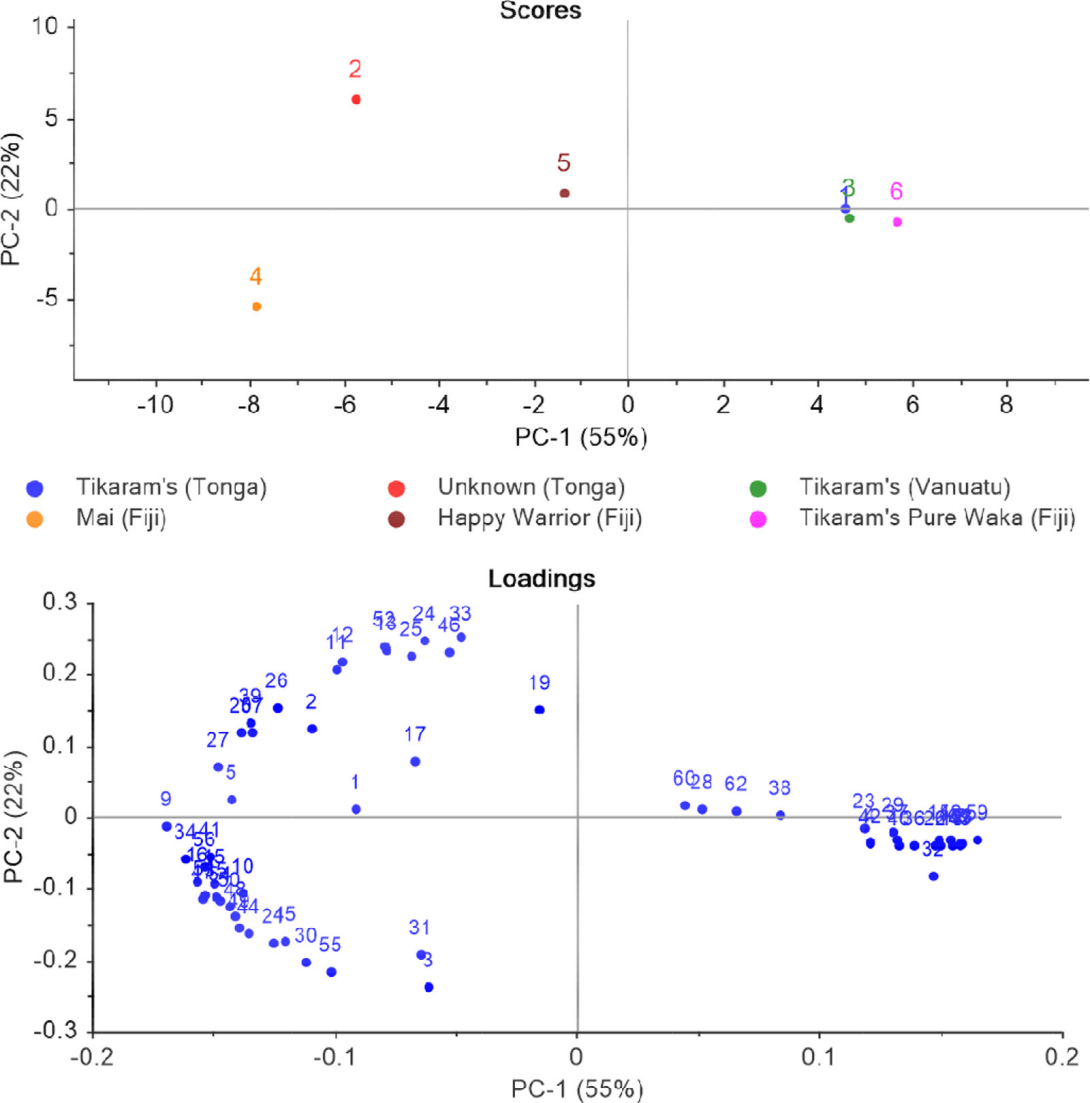
Principal component score plot (top) and loadings plot (bottom) for GC × GC-FID peak area data from kava samples. Data was mean-centered, scaled to standard deviation, and unit vector normalized.

When dealing with VOC profiles for multiple samples, one of the first processing steps many investigators will perform to visualize the structure of the data and monitor pre-processing of the data is principal component analysis (PCA). The highly multivariate nature of the data often means that the number of features (i.e. compounds) far exceeds the number of replicates for each group of samples [11]. PCA is an effective way to initially visualize the data to understand which of the many features identified by GC×GC are important to differentiating one sample from another. Fig. 3 demonstrates the pre-processed data (i.e. using unit-vector normalization, scaling to standard deviation, and mean centering) for the FID peak areas in the dataset. These data preprocessing steps were accomplished to minimize chromatographic noise in the data. The unit-vector normalization allows normalization to the highest peak in the chromatogram, and scaling to standard deviation improves comparability between variables. Both are common pretreatment steps for chromatographic data. The PCA is provided as a means of visualizing the output of the data processing workflow and for comparison by others who use different software packages for statistical processing of the data. In this particular dataset, it was noted that the kava samples from the same commercial retailer were more similar than the kava samples acquired from other retailers. This could be an artifact of the processing of the plant or it could indicate that sample origins are mislabeled on retail packages from which the samples were acquired. However, future work with increased replicates and larger batches will investigate this further. As mentioned previously, this dataset was kept intentionally small in order to provide a step-by-step workflow for processing batch data. This workflow can then be applied to larger batches of data for more comprehensive projects.

The combination of MS and FID data has been seen in other applications in the past, but is rarely used for large batch data for VOC samples and does not have defined batch processing methods. The ability to exploit the selectivity and identification power of the MS data stream with the range and quantitation power of the FID data stream is undoubtedly a value for many applications. Though this dataset represents a small example of batch data for simplicity, it allowed numerous challenges in larger batch data to be identified in the data processing workflow and for methodological parameters to be defined. Users should be conscious of challenges in the data processing workflow for dual channel detection, including the impact that dynamic range and peak width variation may have on pre-processing, and incorporation of representative samples into stencil generation. The transformation of MS stencil to FID data was facilitated by the two-dimensional nature of the plot, and this process drastically reduces the amount of time required for sequencing large MS batch data. The authors present this dataset for other users looking to implement similar methods on VOC samples to facilitate transparency in approaches and improve data integrity for new software tools. It is hoped that the presentation of a full analysis and processing method workflow, from raw data to final results, will assist with the dissemination of GC × GC to new users and improve adoption of GC × GC across an increased number of disciplines.

## Declaration of Competing Interest

The authors declare that they have no known competing financial interests or personal relationships that could have appeared to influence the work reported in this paper.

## References

[1] J.M. Byrne, L.M. Dubois, J.D. Baker, J.-F. Focant, K.A. Perrault, Volatile organic compound data files from six kava (Piper methysticum) samples collected and analyzed by GC×GC-qMS/FID. 2020, Harvard Dataverse, V1. [dataset], DOI: 10.7910/DVN/9APYDN.

[2] J.M. Byrne, L. M. Dubois, J.D. Baker, J.-F. Focant, K.A. Perrault, Methods for sampling, acquisition and data processing of six kava (Piper methysticum) samples collected and analyzed by GC×GC-qMS/FID. 2020, Harvard Dataverse, V1. [dataset] DOI: 10.7910/DVN/OUP5SW.

[3] J.M. Byrne, L.M. Dubois, J.D. Baker, J.-F. Focant, K.A. Perrault, PCA results for six kava (Piper methysticum) samples collected and analyzed by GC×GC-qMS/FID. 2020, Harvard Dataverse, V1. [dataset] DOI: 10.7910/DVN/CEZQOF.

[4] J.M. Byrne, L.M. Dubois, J.D. Baker, J.-F. Focant, K.A. Perrault, Sample reports and alignment of six kava (Piper methysticum) samples collected and analyzed by GC×GC-qMS/FID. 2020, Harvard Dataverse, V1. [dataset] DOI: 10.7910/DVN/ECOTOU.

[5] Dubois L.M., Aczon S., Focant J.-F., Perrault K.A. (2020). Translation of a one-dimensional to a comprehensive two-dimensional gas chromatography method with dual-channel detection for volatile organic compound measurement in forensic applications. Anal. Chem..

[6] Preston B.R., Mcgregor L., Barden D., Column T. (2018). Combining Sorptive Extraction with Two-Dimensional Gas Chromatography for the Flavour Profiling of Milk.

[7] Stilo F., Liberto E., Reichenbach S.E., Tao Q., Bicchi C., Cordero C. (2019). Untargeted and Targeted Fingerprinting of Extra Virgin Olive Oil Volatiles by Comprehensive Two-Dimensional Gas Chromatography with Mass Spectrometry: Challenges in Long-Term Studies. J. Agric. Food Chem..

[8] Reichenbach S.E., Carr P.W., Stoll D.R., Tao Q. (2009). Smart Templates for peak pattern matching with comprehensive two-dimensional liquid chromatography. J. Chromatogr. A.

[9] Reichenbach S.E., Tian X., Tao Q., Ledford E.B., Wu Z., Fiehn O. (2011). Informatics for cross-sample analysis with comprehensive two-dimensional gas chromatography and high-resolution mass spectrometry (GCxGC-HRMS). Talanta.

[10] Perrault K.A., Nizio K.D., Forbes S.L. (2015). A comparison of one-dimensional and comprehensive twodimensional gas chromatography for decomposition odour profiling using inter-year replicate field trials. Chromatographia.

[11] Stefanuto P.-H., Perrault K.A., Stadler S., Pesesse R., LeBlanc H.N., Forbes S.L., Focant J.-F. (2018). GC×GC-TOFMS and supervised multivariate approaches to study human cadaveric decomposition olfactive signatures. Anal. Bioanal. Chem.Bioanal. Chem.Bioanal. Chem.Bioanal. Chem.Bioanal. Chem.Bioanal. Chem..

[12] Showman A.F., Baker J.D., Linares C., Naeole C.K., Borris R., Johnston E., Konanui J., Turner H. (2015). Contemporary Pacific and Western perspectives on ’awa (Piper methysticum) toxicology. Fitoterapia.

